# Multifaceted Mesodiencephalic Triangles: Insights into Hypertrophic Olivary Degeneration and Oculopalatal Tremor Pathophysiology

**DOI:** 10.1007/s12311-025-01903-1

**Published:** 2025-09-04

**Authors:** Jorge C. Kattah, Kavya Moravineni, Eric Eggenberger, Cody Eggenberger, Aasef G. Shaikh, Rodger J. Elble

**Affiliations:** 1 Department of Neurology, University of Illinois, Peoria, IL USA; 2 Department of Neurology, Mayo Clinic, Jacksonville, FL USA; 3 Department of Earth and Environmental Sciences, Florida International University, Florida, FL USA; 4 Department of Neurology, University Hospitals, Case Western Reserve University, Cleveland, OH USA; 5 Department of Biomedical Engineering, Case Western Reserve University, Cleveland, OH USA; 6 Daroff-Dell’Osso Laboratory, Louis Stokes Cleveland VA Medical Center, Cleveland, OH USA; 7 Department of Neurology, Southern Illinois University School of Medicine, Springfield, IL USA

**Keywords:** Tremor, Oculopalatal tremor, Olivary degeneration, Inferior olivary complex, Cerebellum, Brainstem

## Abstract

Oculopalatal tremor is a rare neurological disorder characterized by rhythmic oscillations of ocular and palatal muscles. This phenomenon is commonly associated with hypertrophic degeneration of the inferior olive due to loss of GABAergic cerebello-olivary fibers. Oculopalatal tremor highlights the complex interplay between cerebellar, mesodiencephalic, and olivary networks. The principal, medial accessory, and dorsal accessory subnuclei of the inferior olive exhibit rhythmic subthreshold oscillations that are hypothesized to play an important role in oculopalatal tremor. However, delayed tremor onset and variability in hypertrophic olivary degeneration challenge the hypothesis of olivary oscillation as the principal cause of tremorogenesis. A plausible alternative hypothesis is that tremorogenic oscillation emerges from maladaptive cerebellar network plasticity in response to the loss of physiologic climbing fiber activity. The anatomy and physiology of the fastigiobulbar connections are compatible with the clinical characteristics of oculopalatal tremor syndrome, and clinicopathological correlations suggest that the fastigiobulbar pathway is necessary for oculopalatal tremor. Latent brainstem oscillators, released by cerebellar dysfunction, have been proposed but do not explain the anatomical distribution of oscillation. Advanced imaging and computational models have provided insights into possible mechanisms of oscillation but underscore the need for further studies, particularly in a suitable animal model, which does not exist.

**Supplementary Information:**

The online version contains supplementary material available at 10.1007/s12311-025-01903-1.

## Introduction

**Fig. 1:**
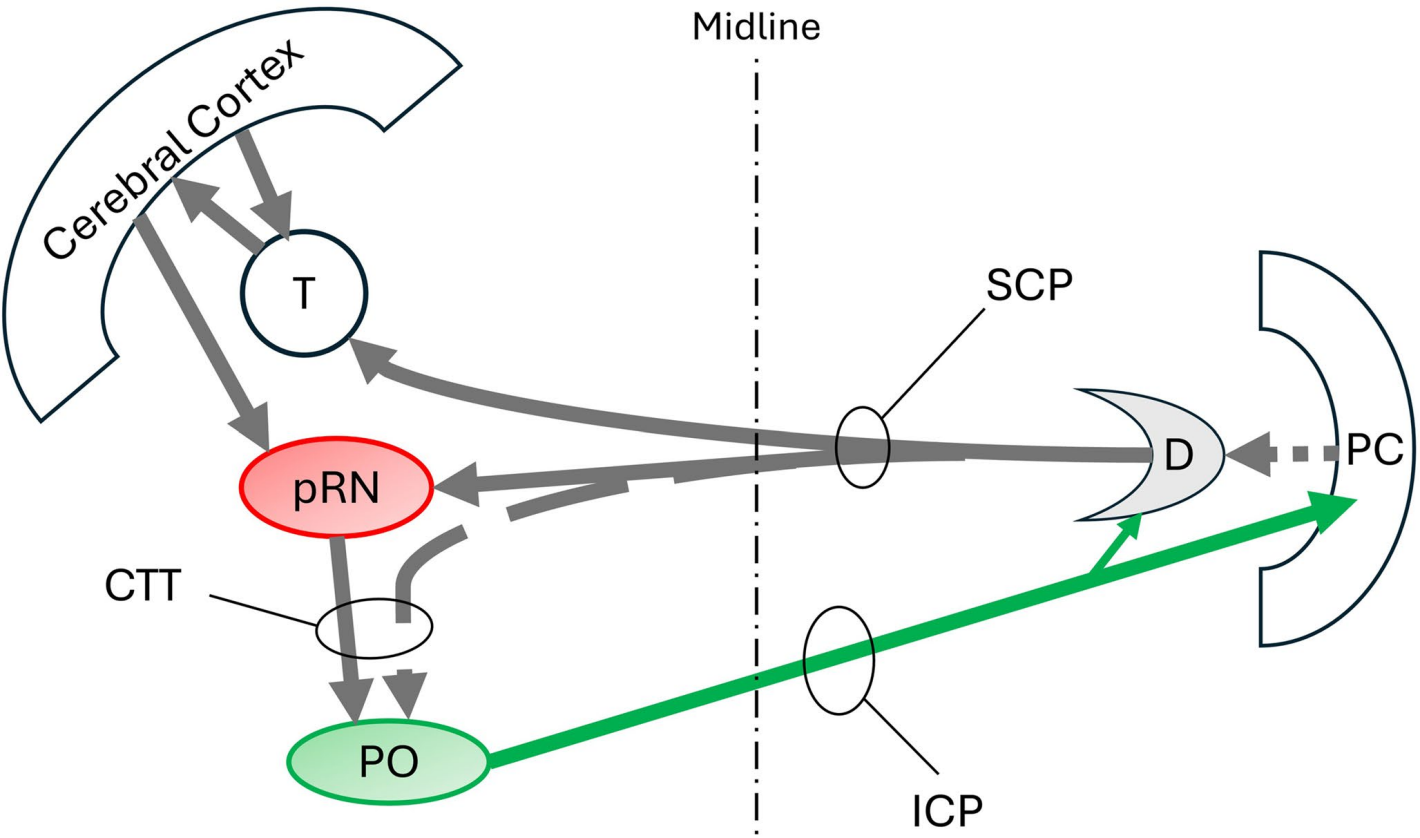
Schematic diagram of the dentato-rubro-olivary loop or Guillain-Mollaret triangle. CTT: central tegmental tract, D: dentate, ICP: inferior cerebellar peduncle, PC: Purkinje cells, PO: principal olive, pRN: parvocellular red nucleus, SCP: superior cerebellar peduncle, T: thalamus. Solid grey and green arrows are glutaminergic. Broken arrows are GABAergic

The 2-Hz ocular, pharyngeal and laryngeal “nystagmus” described in 1886 by Spencer [1] was subsequently called oculopalatal myoclonus but is now classified as a tremor [2]. In 1938, Guillain reviewed seminal clinicopathological correlations that were conducted in collaboration with Mollaret and Bertrand and by others [3]. He noted that the 1–3 Hz palatal tremor is frequently associated with repetitive to-and-fro eye movements, often called “pendular” nystagmus, at a similar frequency and less commonly associated with periodic contractions of the face, tongue, larynx, diaphragm, intercostal muscles, torso and extremities [3]. The periodic contractions in various locations were usually synchronous and were associated with lesions in the dentato-rubro-olivocerebellar loop, which is now commonly called the Guillain-Mollaret triangle (Fig. 1). Hypertrophic degeneration of the inferior olives was often found at autopsy, and olivary degeneration was hypothesized to play an important role in tremor pathophysiology. Patients with unilateral tremor usually had hypertrophic degeneration of the contralateral olive. In addition to the periodic muscle contractions of this tremor, there is also time-locked periodic inhibition of muscle contraction in the extremities that is asymptomatic [4, 5]. Therefore, this movement disorder can be much more widespread than clinically appreciated. Here we refer to these periodic phenomena collectively as oculopalatal tremor syndrome, recognizing that the eyes and palate may oscillate individually and in combination with periodic muscle contraction and inhibition elsewhere.

The association between hypertrophic olivary degeneration and oculopalatal tremor syndrome has been confirmed by numerous investigators, and this association is now visible with magnetic resonance imaging [4, 6, 7]. Patients without olivary hypertrophy usually exhibit isolated palatal tremor with an associated ear click due to periodic opening and closure of the eustachian tube [4]. This “essential” palatal tremor is idiopathic and often functional, and it clearly differs from the oculopalatal tremor syndrome associated with hypertrophic olivary degeneration and other discernible pathology in the brainstem or cerebellum (i.e., “symptomatic” palatal tremor) [4, 8–11]. Symptomatic palatal tremor is a misleading term because the palatal tremor is usually asymptomatic (rarely causes an ear click) and is missed without a careful neurologic exam [12]. However, the pendular nystagmus is commonly associated with oscillopsia [12]. Thus, oculopalatal tremor syndrome is an important sign of damage to the cerebello-olivary pathway, and the pathophysiology of this rare movement disorder is the subject of this review.

Multiple homologous mesodiencephalic triangles.

The inferior olive has three main subdivisions: the principal olive, dorsal accessory olive and medial accessory olive. In addition, there are four minor subdivisions: the dorsal cap of Kooy, the β subnucleus, the ventrolateral outgrowth, and the dorsomedial cell column [13]. The principal olive is by far the largest subnucleus in man [14, 15], and hypertrophic degeneration of the principal olive is readily visible with MRI [6, 7]. Neurons of the principal olive, dorsal accessory olive and medial accessory olive feature rhythmic subthreshold membrane voltage oscillations due to T-type calcium channels (CaV3.1) and hyperpolarization-activated potassium currents [16, 17].

Lapresle and Hamida performed meticulous clinicopathological correlations demonstrating that olivary hypertrophy is a degenerative response to the loss of dentato-olivary fibers [18]. Dentate projects in a topographical manner to the contralateral principal olive, and damage at any point in the dentato-olivary pathway produces hypertrophic degeneration in areas of the principal olive that are consistent with normal anatomical topography. Dentato-olivary fibers are now known to make GABAergic synapses on the electrotonic gap junctions between dendrites of olivary neurons [17]. Dentate also projects to the contralateral parvocellular red nucleus (glutaminergic), which projects to the ipsilateral principal olive (glutaminergic) [15]. This disynaptic excitatory pathway is nearly always affected by lesions in the monosynaptic inhibitory pathway, so both pathways must be considered in discussions of oculopalatal tremor syndrome. These parallel inhibitory and disynaptic excitatory pathways control the timing of olivary complex spike production [19].

The parvocellular red nucleus is the largest member of several mesodiencephalic nuclei that provide junctures for coordinating the influence of specific regions of cerebral cortex and cerebellum on the activity of specific olivary subnuclei. Knowledge of this complex anatomy comes mainly from studies of lower mammals and is reviewed elsewhere [20]. The anatomy and physiology of the dentato-rubro-olivary loop (i.e., Guillain-Mollaret triangle) are not sufficient to explain the oculopalatal tremor syndrome. Loops involving all cerebellar nuclei must be considered.

**Fig. 2:**
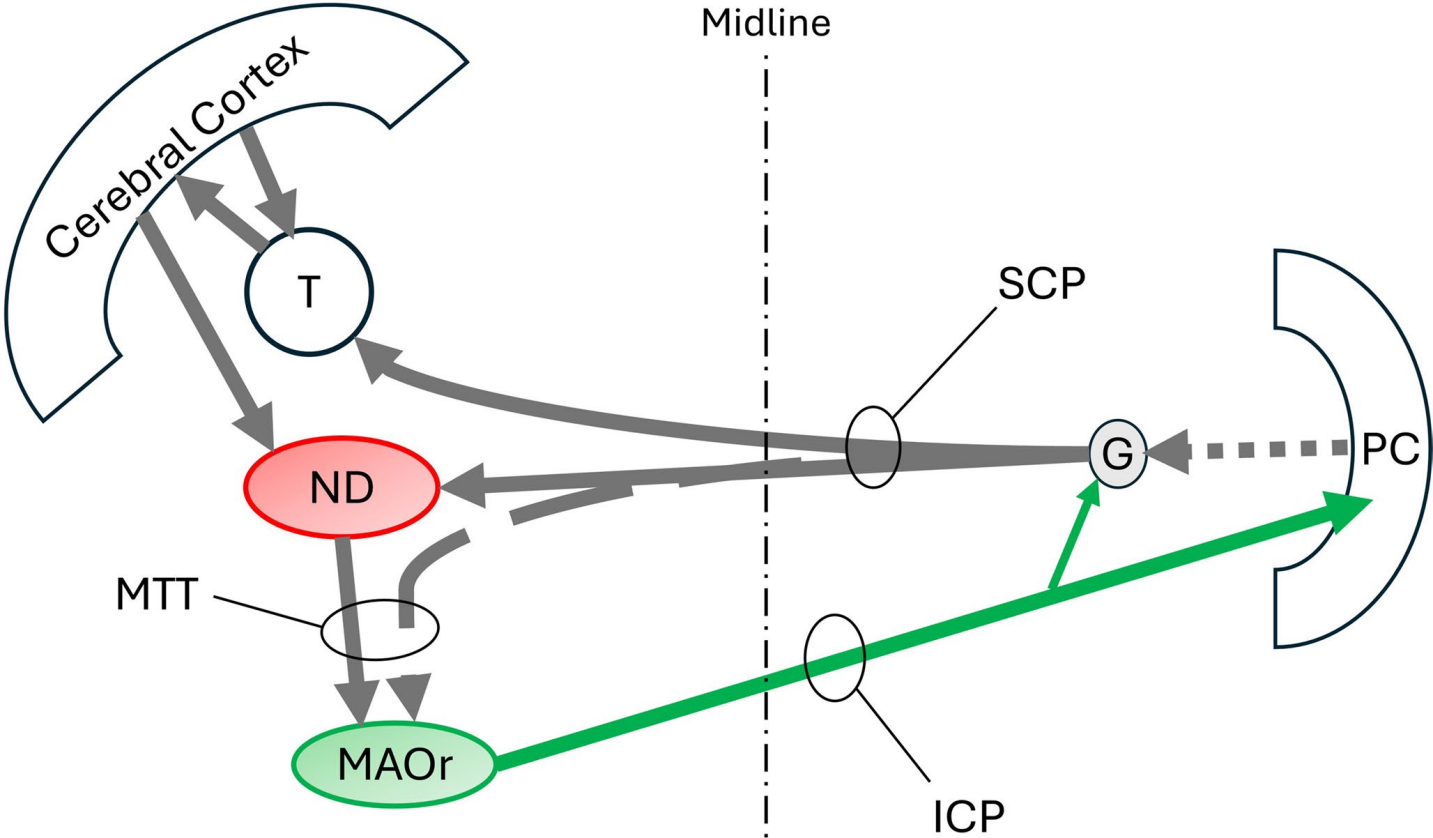
Mesodiencephalic triangle involving the nucleus of Darkschewitsch (ND). DMCC: dorsomedial cell column of the inferior olive, G: globose nucleus, ICP: inferior cerebellar peduncle, MAOr: rostral medial accessory olive, MTT: medial tegmental tract, PC: Purkinje cells, SCP: superior cerebellar peduncle, T: thalamus. Solid grey and green arrows are glutaminergic. Broken arrows are GABAergic

The posterior interpositus (globose nucleus) forms a triangular network with the nucleus of Darkschewitsch, rostral medial accessory olive, and olivary dorsomedial cell column (Fig. 2) [21, 22]. This network participates with cerebral frontal eye fields in oculomotor control [21].

The anterior interpositus (emboliform nucleus) and dorsal accessory olive are involved in cerebellar feedback and feedforward motor control and do not form a network with a mesodiencephalic nucleus [23]. This may explain why hypertrophic degeneration of the dorsal accessory olive is not observed [22].

**Fig. 3:**
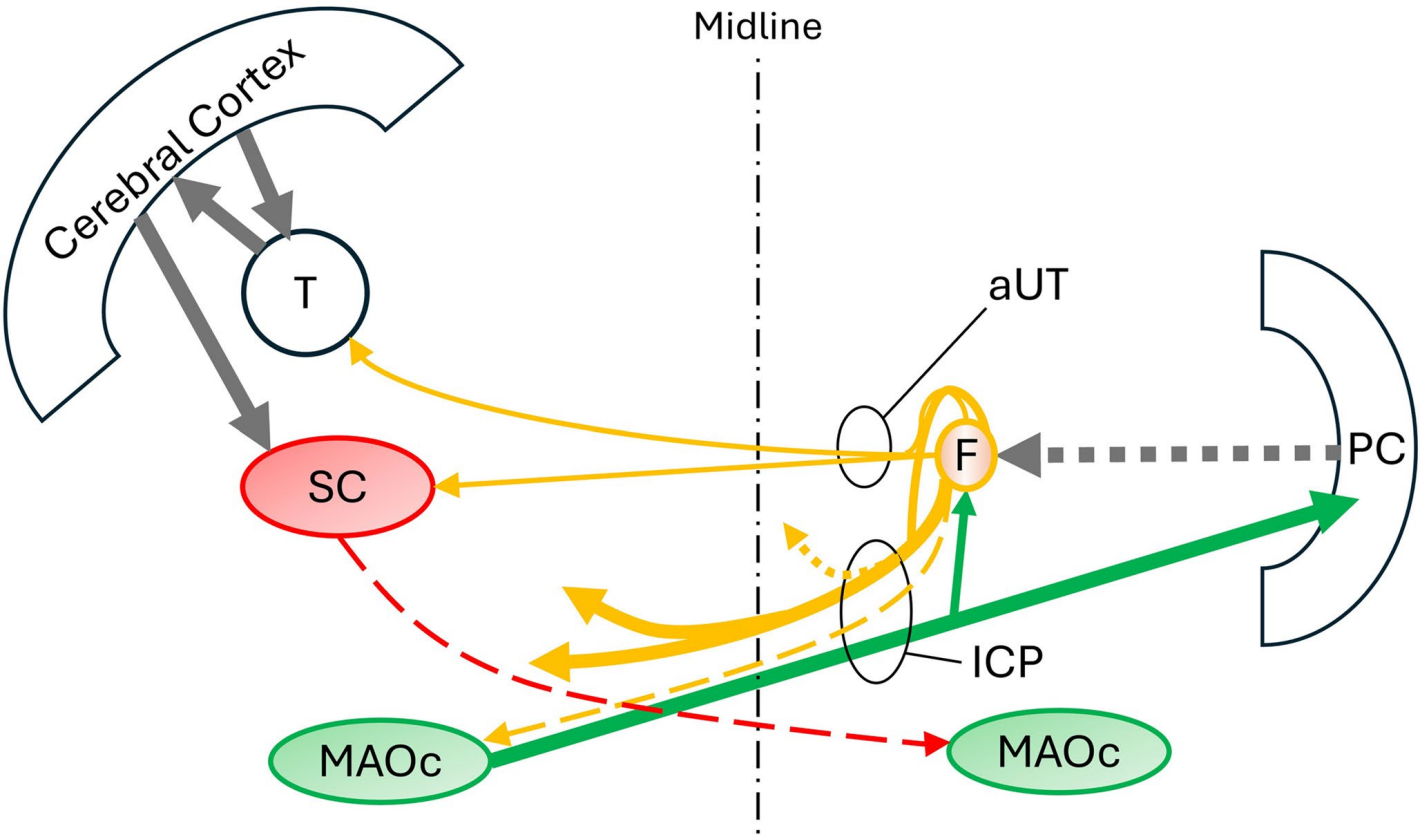
Fastigial (F) connections with the caudal medial accessory olive (MAOc) and deep tectal neurons of superior colliculus (SC). aUT: ascending branch of the fastigial uncinate fasciculus, ICP: inferior cerebellar peduncle, PC: Purkinje cells, T: thalamus. Solid grey, green and orange arrows are glutaminergic. Broken arrows are GABAergic. Dotted orange arrow is glycinergic. The projection from fastigium to the medial accessory olive is unestablished in humans and is predominantly excitatory, not inhibitory, in rodents

Fastigio-olivary projections in man are still questioned and are not mentioned in some reviews [22, 24]. By contrast, a projection from the caudal medial accessory olive to the fastigial nucleus is widely acknowledged. When reciprocal connections are described, the olivofastigial projection is always reported as being disproportionately larger [25]. Two studies in monkeys revealed no projections from the fastigial nucleus to any part of the inferior olivary complex [14, 15]. However, three subsequent studies found that the caudal fastigial nucleus forms a network with deep tectal neurons of the superior colliculus and caudal medial accessory olive [26–28]. This network participates in oculomotor control and the coordination of head and eye movements [26, 29]. Studies in mice found that this fastigial network differs from the Guillain-Mollaret triangle in the following important ways. The small projection from fastigium to the contralateral caudal medial accessory olive is predominantly excitatory, not inhibitory, and the deep tectal neurons of the superior colliculus are predominantly inhibitory to the caudal medial accessory olive, not excitatory [30, 31]. Furthermore, these tectal neurons project to the contralateral caudal medial accessory olive, not ipsilateral (Fig. 3) [30]. In other words, the fastigial nucleus does not participate in a mesodiencephalic triangle consisting of parallel inhibitory input and disynaptic excitatory input to the contralateral olive.

Hypertrophic change occurs in the rostral but not caudal medial accessory olive following contralateral hemi-cerebellectomy in cats [32]. Boesten and Voogd concluded that hypertrophic degeneration is restricted to those olivary subnuclei that participate in cerebello-mesodiencephalic-olivary loops, such as those in Figs. 1 and 2 [33].

A review of published cases of olivary hypertrophy found that only 33.8% of patients exhibited palatal tremor [34]. Furthermore, the olivary subnuclei receiving input from the interposed and fastigial nuclei are too small to be discerned with MRI, yet they are more likely to be involved in tremorogenesis, given their known anatomy and physiology. Thus, clinicopathological correlations of oculopalatal tremor with olivary hypertrophy using MRI could be misleading.

The nucleus prepositus hypoglossi receives Purkinje cell input from the flocculus and nodulus of the cerebellum and is therefore viewed as an extension of the cerebellar nuclei. The fastigial nucleus also projects to nucleus prepositus hypoglossi [35]. This nucleus has been suggested as a possible source of ocular oscillation in oculopalatal tremor syndrome [36]. Nucleus prepositus hypoglossi sends GABAergic fibers to the gap junctions among neurons in dorsal cap of Kooy bilaterally, and it sends excitatory fibers to mesodiencephalic neurons that project to the dorsal cap of Kooy [37]. Neurons of dorsal cap of Kooy and the ventrolateral outgrowth do not have the subthreshold oscillatory properties of principal and accessory olivary subnuclei [16, 17], so any participation of the nucleus prepositus hypoglossal network in the pathogenesis of oculopalatal tremor could be interpreted as evidence against the hypothesis that the inferior olive is the primary source of oscillation. The nucleus prepositus hypoglossi also connects with the β subnucleus of the inferior olive [35], but we could find no electrophysiologic studies of this subnucleus.

Is the inferior olive the source of oscillation in oculopalatal tremor?

The 1–10 Hz oscillatory properties of the inferior olive neurons and the electrotonic gap junctions between neuronal dendrites are well documented and widely emphasized in discussions of olivocerebellar physiology [17, 38]. The timing of sequential monosynaptic GABAergic and disynaptic glutaminergic input to olivary neurons affects the transient entrainment of subthreshold oscillations in subpopulations of olivary neurons, thereby controlling the likelihood and timing of complex spike generation [19].

The mesodiencephalic triangles serve to deliver timely and appropriately distributed complex spikes to the cerebellum at 1–3 per second [19]. The aperiodic low-frequency firing patterns of climbing fibers bear little or no obvious relationship to the kinematics or kinetics of movement [39–41], but complex spikes are more likely to occur when there is a novel unexpected sensory stimulus or change in behavioral conditions [38]. These observations are consistent with the view that the inferior olive participates in the timing or coordination of motor adaptation and learning [41, 42].

The prevailing hypothesis is that oculopalatal tremor emerges from the oscillatory properties of the inferior olive. Hypertrophic olivary degeneration is a response of olivary neurons to the loss of GABAergic afferent fibers from the cerebellar nuclei [18, 43, 44]. These fibers inhibit electrotonic dendrodendritic synapses (gap junctions) among olivary neurons [45]. Therefore, GABAergic deafferentation of the olive could pathologically increase coupling among olivary neurons and thereby facilitate oscillatory entrainment among olivary neurons. This increased coupling arguably should occur immediately, and palatal tremor should begin immediately if increased coupling is the primary cause of palatal tremor. However, palatal tremor typically begins 1–2 months after the cerebellar or brainstem ictus, and palatal tremor persists for years, long after the inferior olives are largely devoid of neurons [18, 46, 47]. The delay in tremor onset suggests that tremorogenic changes in olivocerebellar networks occur in response to the initial etiologic event, which is most commonly vascular. The persistence of oculopalatal tremor suggests that cerebellar oscillation is not simply driven by olivary oscillation.

Harmaline is a drug that enhances synchronous oscillation in the inferior olive and produces a generalized musculoskeletal tremor, but it has no effect on the eyes [48]. This experimental observation is strongly at odds with the hypothesis that the inferior olive is the source of tremorogenic oscillation in oculopalatal tremor.

There is no animal model of oculopalatal tremor. Neither olivary hypertrophy nor palatal tremor occurred in rats 8 months after superior cerebellar peduncle transection [49]. Perhaps a longer period of observation is required, and species variability in the olivary response to GABAergic deafferentation is also possible. Cerebellectomy in cats produces hypertrophic degenerative change in olivary neurons, but no rhythmic olivary discharge or oculopalatal tremor has been reported [32, 33, 50]. It is possible therefore that some portion of the cerebellum must be preserved for oculopalatal tremor to occur. In humans, ocular tremor appears to require the preservation of the cerebellar flocculus [51], and oculopalatal tremor has never been described in a patient with destruction of the inferior cerebellar peduncle [18]. These observations are consistent with the hypothesis that the flocculus and fastigial nucleus are necessary for oculopalatal tremor to occur.

The lack of a good animal model plagues all tremor research and explains why the source of tremorogenic oscillation is still unknown for all forms of tremor except physiological tremor [52]. Lesions in the Guillain-Mollaret triangle have been studied in monkeys, but not with the goal of producing oculopalatal tremor. Larochelle and workers systematically lesioned the three arms of the Guillain-Mollaret triangle and surprisingly produced no sustained tremor [53]. However, any lesion or combination of lesions produced 4–6 Hz postural tremor when the animals were injected with harmaline [53]. Larochelle and workers concluded that “the released nervous centres could involve either the cerebellar cortex and/or the fastigial nuclei and the efferent connections to the lower brain stem; or else, they may involve certain upper brain stem structures that are normally under the influence of the ascending cerebellofugal fibres”. Harmaline has long been known to enhance olivary rhythmicity [54, 55], but the tremorogenic effects of harmaline are not limited to the olive [56].

Oculopalatal tremor differs from most other forms of tremor in that the periodic muscle excitation and inhibition in oculopalatal tremor syndrome is usually asymptomatic, except for the oscillopsia associated with the pendular nystagmus (i.e., ocular tremor) [12, 57]. This must be a clue to the olivocerebellar anatomy and physiology of oculopalatal tremor. Electrical stimulation of the inferior olive produced no movements or modification of movement in cats [42], suggesting that synchronous oscillation of olivary neurons is not likely to have gross motor effects.

It is now known that unilateral hypertrophic olivary degeneration does not correlate strictly with the laterality of oculopalatal tremor [58]. This observation has been interpreted as evidence against the olivary hypothesis of oculopalatal tremor. However, this could also be due to a limited sensitivity of MRI to mild hypertrophic change [59]. Bilateral brainstem projections from the fastigial nucleus could also play a role [21]. If the inferior olive is the source of oculopalatal oscillation, fastigiobulbar fibers must mediate the distribution of olivary oscillation to the brainstem because olivary fibers project solely to the deep cerebellar nuclei and Purkinje cells of the cerebellar cortex. Fastigial efferents are glutaminergic, GABAergic and glycinergic [60]. The fastigial nucleus projects widely to the brainstem in a manner that is independent of the dentate and interposed projections in the superior cerebellar peduncle (Fig. 3) [15]. These fastigial projections participate in the control of eye movements, respiratory movements, swallowing, locomotion, and balance and are consistent with the anatomical distribution of oculopalatal tremor [24, 61]. Thus, fastigiobulbar connections are compatible with the clinical characteristics of oculopalatal tremor syndrome, and clinicopathological correlations suggest that the fastigiobulbar pathway is necessary for oculopalatal tremor.

The delayed onset of olivary hypertrophy and oculopalatal tremor suggests that maladaptive change in olivocerebellar network occurs after interruption of cerebello-olivary GABAergic fibers. Shaikh and coworkers [62] hypothesized that subpopulations of olivary neurons become abnormally synchronized at 1–3 Hz due to loss of GABAergic inhibition of gap junctions between the dendrites of olivary neurons and that the cerebellum responds to this abnormal olivary activity by “learning” to oscillate at 1–3 Hz. The feasibility of this hypothesis was demonstrated with a computer model [62]. This model explains the delay in onset of oculopalatal tremor and the long-lasting persistence of tremor after the inferior olive is largely depleted of neurons. Moreover, greater olivary synchrony produced by loss of cerebello-olivary inhibitory fibers could produce excitotoxic death of Purkinje cells [63], and this and similar phenomena could also play a role in the delayed onset of oculopalatal tremor.

Cerebellar oscillation hypothesis.

An observation against the olivary oscillation hypothesis is that oculopalatal tremor occurs in patients with olivary destruction due to adult-onset Alexander disease and medullary stroke, but with no history of intervening olivary hypertrophy [64]. Olivary hypertrophy has been reported in adult-onset Alexander disease [65, 66], but severe olivary atrophy is most common [67, 68]. It is possible that olivary hypertrophy is missed in some cases due to imperfect sensitivity of MRI or the temporary nature of olivary enlargement and T2 hyperintensity [59]. It is also possible that some combinations of afferent and efferent fiber destruction are more likely to produce atrophy without intervening hypertrophy.

An alternative hypothesis is that tremorogenic oscillation stems from the loss of normal climbing fiber activity rather than olivary oscillation per se. It is possible that the cerebellum develops abnormal tremorogenic oscillation in response to the loss of normal climbing fiber activity. This alternative hypothesis is a modification of the cerebellar learning hypothesis of Shaikh and colleagues in which increased oscillatory climbing fiber activity is the initial event [62].

There is experimental evidence that the cerebellum can develop abnormal oscillatory behavior when climbing fibers are blocked or destroyed. Acute blockade of olivo-cerebellar activity in mice produced dystonic muscle contractions and tremor due to oscillatory firing in interposed neurons [69]. Chronic destruction (2 years) of the inferior olives in rats with 3-acetylpyridine produced an ataxia syndrome with 3–6 Hz tremor [70]. This correlated with abnormal rhythmic bursting activity in approximately half of recorded Purkinje cells and in all nuclear cells [70]. Neurons of the cerebellar nuclei have oscillatory properties similar to those of olivary neurons [71], and amplification of oscillation at the nuclear level can be explained by the fact that relatively large numbers of Purkinje cells converge on glutaminergic and GABAergic cerebellar nuclear cells (ratio > 10:1) [72].

Pharyngeal contractions at 2–5 Hz were observed in cats following surgical ablation of the inferior olives [73] but were not observed in cats following cerebellectomy, despite the occurrence of variable hypertrophic change in olivary neurons [32, 33]. These observations are consistent with the hypothesis that oculopalatal tremor is mediated by efferent fibers in the inferior cerebellar peduncle from the fastigial nucleus and possibly from Purkinje cells in the vestibulo-ocular cerebellar cortex.

Destruction of the inferior olive may have downstream transsynaptic effects on the cerebellum. Purkinje cells undergo degenerative change in rats [74] and in cats [75], and the cerebellar nuclei develop low-frequency bursting activity [70]. Horn and colleagues observed progressive ataxia beginning 13 days after destruction of the inferior olive with kainic acid injection [75].

Fluorodeoxyglucose-PET studies have found hyperactivity in the hypertrophic olivary nuclei of some patients with oculopalatal tremor [58, 76, 77] but not in others [77–79]. One PET study found hyperactivity in the contralateral thalamus [79], which could be explained by fastigiothalamic projections [22, 61]. Another study demonstrated a reduction in glucose utilization in the inferior vermis but not the hypertrophic olive after successful treatment of ocular tremor with clonazepam [58], and this observation suggests that the cerebellar cortex plays a pivotal role in tremorogenesis.

Oculopalatal tremor has been treated with gabapentin, memantine and clonazepam, but the clinical response has been too modest, inconsistent and anatomically nonspecific to be used as a tool for deciphering olivary and cerebellar origins of oscillation [57]. However, a fairly sound pharmacodynamic rationale exists for these drugs and others in the treatment of oculopalatal tremor, as summarized in Table 1. In an uncontrolled study, gabapentin and memantine each reduced the rhythmicity of ocular tremor in patients with oculopalatal tremor [80]. Gabapentin binds to the α2δ−1 and − 2 subunits of presynaptic voltage-gated calcium channels and can thereby reduce excitatory neurotransmitter release and neuronal excitability [81]. Inferior olivary neurons predominantly express the α2δ−1 subunit, and Purkinje neurons express the α2δ‑2 subunit at the climbing fiber-Purkinje cell synapse [82]. Memantine is a low-affinity N-methyl-D-aspartate (NMDA) receptor antagonist that blocks excess calcium influx through glutaminergic NMDA channels, primarily when overactivated, thus potentially protecting cerebellar and olivary neurons from excitotoxic damage [83, 84]. A neuroprotective effect of memantine was demonstrated in rats with harmaline-induced tremor, even though there was little reduction in tremor [84]. Memantine could have a similar neuroprotective effect during the pathogenesis of oculopalatal tremor. Abundant GABA_A_-benzodiazepine receptors are found in the inferior olive and cerebellum [85–87], providing a rationale for benzodiazepines in suppressing neuronal oscillation and excitability through enhanced GABAergic inhibition [88]. Finally, Cav3.1 T-type voltage-dependent calcium channels interact with hyperpolarization-activated cyclic nucleotide-gated isoform 1 (HCN1) sodium/potassium channels to produce resonant oscillation in olivary neurons [89]. Cav3.1 and HCN1 channels are also present in the cerebellar cortex and deep cerebellar nuclei [90–92]. Cav3.1 is potentiated by the metabotropic glutamate receptor 1 [90], providing yet another potential therapeutic target.

**Table 1:** Potential Pharmacologic targets for the treatment of oculopalatal tremor

Agent	Receptor binding	Neuroanatomical targets	Potentially beneficial pharmacodynamic effect
Gabapentin	α2δ−1 and − 2 subunits of presynaptic voltage-gated calcium channels	Inferior olive Cerebellum	Reduced excitability of neurons participating in tremorogenic oscillation
Memantine	Low-affinity N-methyl-D aspartate (NMDA) receptor antagonist	Inferior olive Cerebellum	Reduced excitability of oscillating neurons. Reduced excitotoxic neuronal damage
Benzodiazepines	GABA_A_-benzodiazepine receptors	Inferior olive Cerebellum	Enhanced GABAergic inhibition of oscillating neurons
T-type calcium channel blockers	Cav3.1 T-type calcium channels	Inferior olive Cerebellum	Reduced resonant oscillation in olivary neurons Reduced bursting in Purkinje cells and deep cerebellar nuclei
hyperpolarization-activated cyclic nucleotide-gated (HCN) channel blockers	HCN isoform 1 channels	Inferior olive Cerebellum	Reduced resonant oscillation in olivary neurons Reduced bursting in Purkinje cells and deep cerebellar nuclei

Release of latent brainstem oscillators.

The cerebellar nuclei project to many areas of the brainstem. Dentate projects to the contralateral parvocellular red nucleus, principal olive, and nucleus reticularis tegmenti pontis [15]. The interposed and fastigial nuclei project more widely to the brainstem, and many projections are inhibitory [60, 61, 93]. Therefore, it is conceivable that latent brainstem oscillators are released by damage to cerebellar efferent pathways. However, there is no convincing evidence for this hypothesis in animal studies, and this hypothesis does not readily explain how rhythmic muscle contraction and inhibition in widespread locations is usually synchronous in oculopalatal tremor syndrome.

Nearly 100 years ago, Stern noted that palatal tremor frequency, rhythmicity, and presence during sleep resembles the respiratory movements of gill breathers in fish and amphibian larvae [94]. According to this hypothesis, latent oscillation in the inspiratory preBötzinger nucleus and lower cranial nerve motoneurons is released or facilitated by impaired cerebello-olivary function, producing oscillation in branchial arch muscles. However, this mechanism does not explain the commonly associated ocular tremor and the less commonly associated tremors in other anatomical locations, such as the face and limbs. The fastigial nucleus projects to the nucleus gigantocellularis, the nucleus solitarius and the ambiguous nuclei, which project to the preBötzinger complex, but there is no direct cerebellar projection to preBötzinger neurons [95]. Nevertheless, the rostral fastigial nucleus is clearly involved in the coordination of breathing with voluntary oromotor activity such as licking and swallowing [96], so fastigial damage or oscillation might affect the muscles involved in these activities.

Summary and conclusions.

The primary origin of tremorogenic oscillation in oculopalatal tremor syndrome is still uncertain. Mesodiencephalic triangles function to purposefully select and time the activation of climbing fibers to the cerebellum. This is accomplished with brief oscillatory entrainment of olivary neurons through the coordinated action of GABAergic cerebello-olivary fibers with glutaminergic cerebello-mesodiencephalic-olivary fibers. The prevailing hypothesis of oculopalatal tremor is that damage to one or more mesodiencephalic triangles produces sustained oscillation in the corresponding subnuclei of the inferior olive, in conjunction with hypertrophic olivary degeneration. However, acute and degenerative destruction of the inferior olive, rather than hypertrophic olivary degeneration, has been observed in a small number of patients with oculopalatal tremor. Therefore, it is possible that the loss of physiologic climbing fiber activity, rather than oscillation per se, leads to maladaptive tremorogenic cerebellar behavior: i.e., sustained oscillation rather than timely purposeful transient oscillation. According to this cerebellar hypothesis, maladaptive oscillation in the fastigial nucleus is transmitted to brainstem nuclei that control eye movement, bulbar musculature, and muscle tone. Even if the inferior olive is the primary source of oscillation, the fastigiobulbar pathways are still necessary for oculopalatal tremor [55].

An animal model of oculopalatal tremor is needed to resolve these hypotheses. A suitable animal model would enable the recording of olivary and cerebellar neuronal activity during different stages of hypertrophic olivary degeneration, and the effects of intervention with pharmacotherapy, stereotactic lesions and neuronal stimulation could be explored. Cerebellar resection produced hypertrophic olivary degeneration in cats, but oculopalatal tremor was not observed [32, 33, 50]. Selective transection of the superior cerebellar peduncle is more likely to produce oculopalatal tremor because the fastigial nucleus and flocculus would be preserved. Ten monkeys were examined for 3–5 months following transection of the superior cerebellar peduncle, but olivary hypertrophy and oculopalatal tremor were not reported [97]. However, it is unclear how carefully the monkeys were examined for these phenomena. Nevertheless, the cat model with superior cerebellar peduncle transection should be explored before moving to primates. The pursuit of an animal model might seem unjustified for a tremor syndrome that is largely asymptomatic. However, the study of oculopalatal tremor has provided important insight into olivo-cerebellar physiology, which is relevant to all tremor disorders.

## Supplementary Information

Below is the link to the electronic supplementary material.

ESM 1 (JPG 456 KB)

ESM 2 (JPG 444 KB)

ESM 3 (JPG 501 KB)

## Data Availability

No datasets were generated or analysed during the current study.
