# Supplementary figures and images for: Multifaceted Mesodiencephalic Triangles: Insights into Hypertrophic Olivary Degeneration and Oculopalatal Tremor Pathophysiology
Source: Cerebellum. 2025 Sep 4;24(5):151. doi: 10.1007/s12311-025-01903-1 (PMC12411318; doi:10.1007/s12311-025-01903-1)

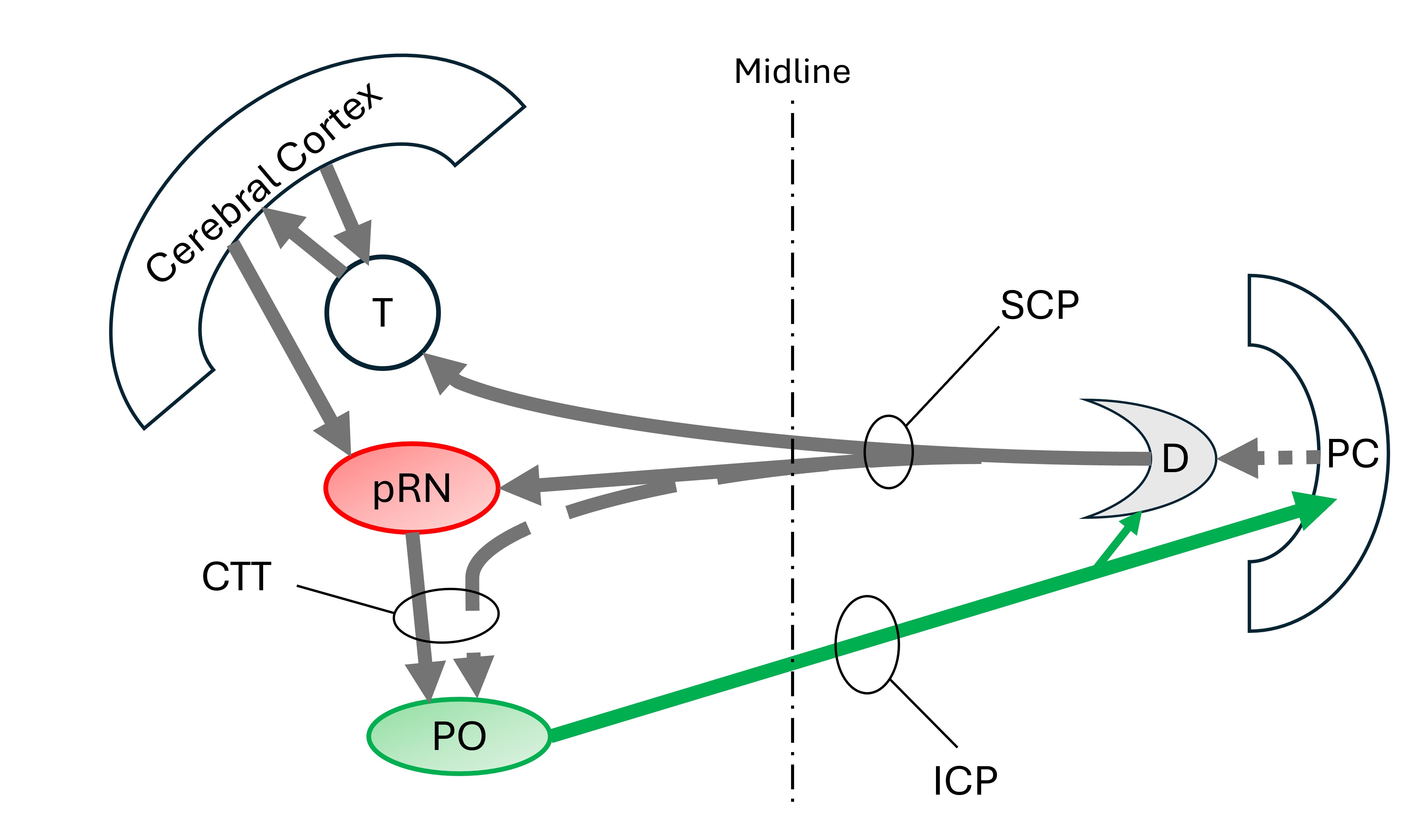

Supplement: Supplementary file 1 — (JPG 456 KB) [file 12311_2025_1903_MOESM1_ESM.jpg]

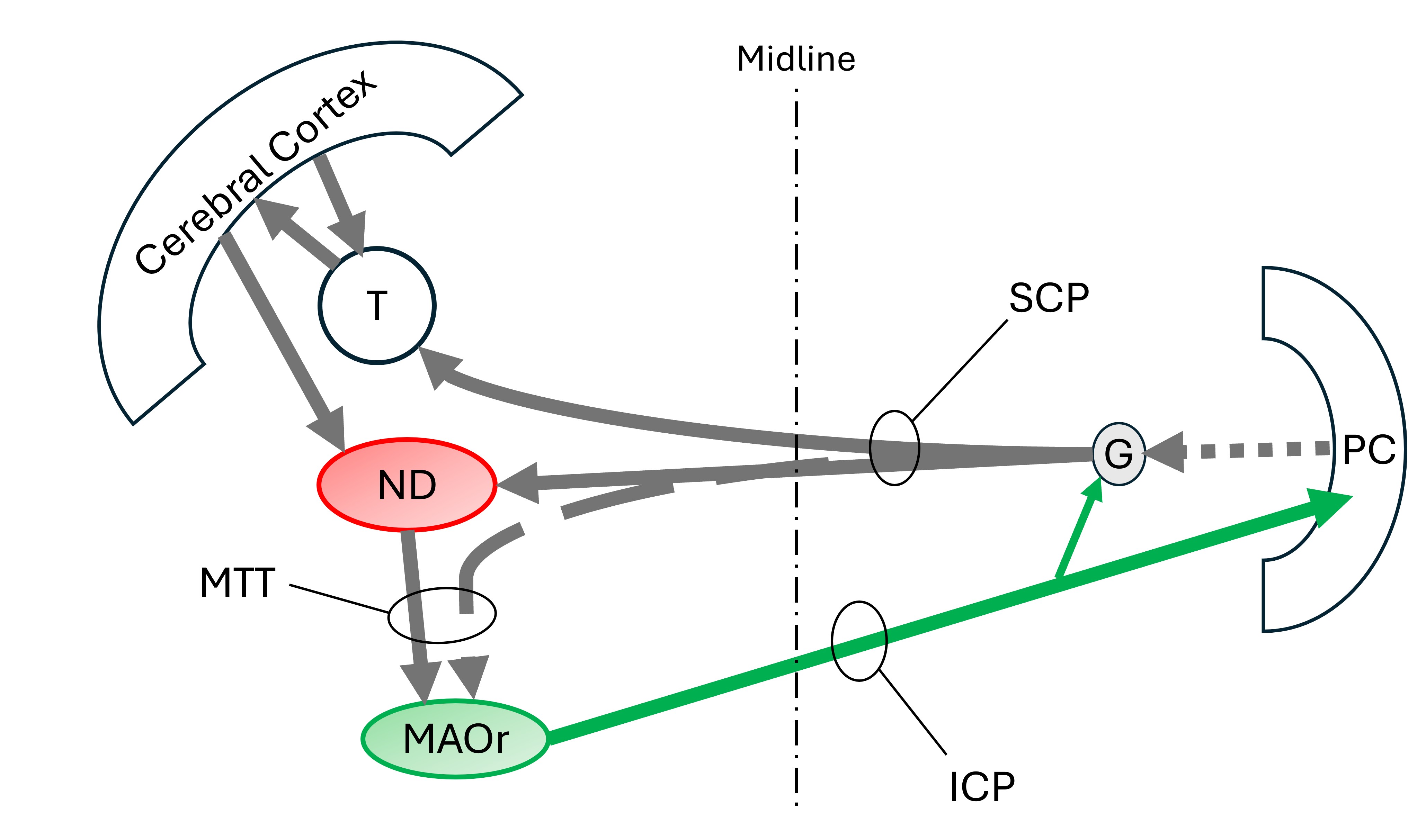

Supplement: Supplementary file 2 — (JPG 444 KB) [file 12311_2025_1903_MOESM2_ESM.jpg]

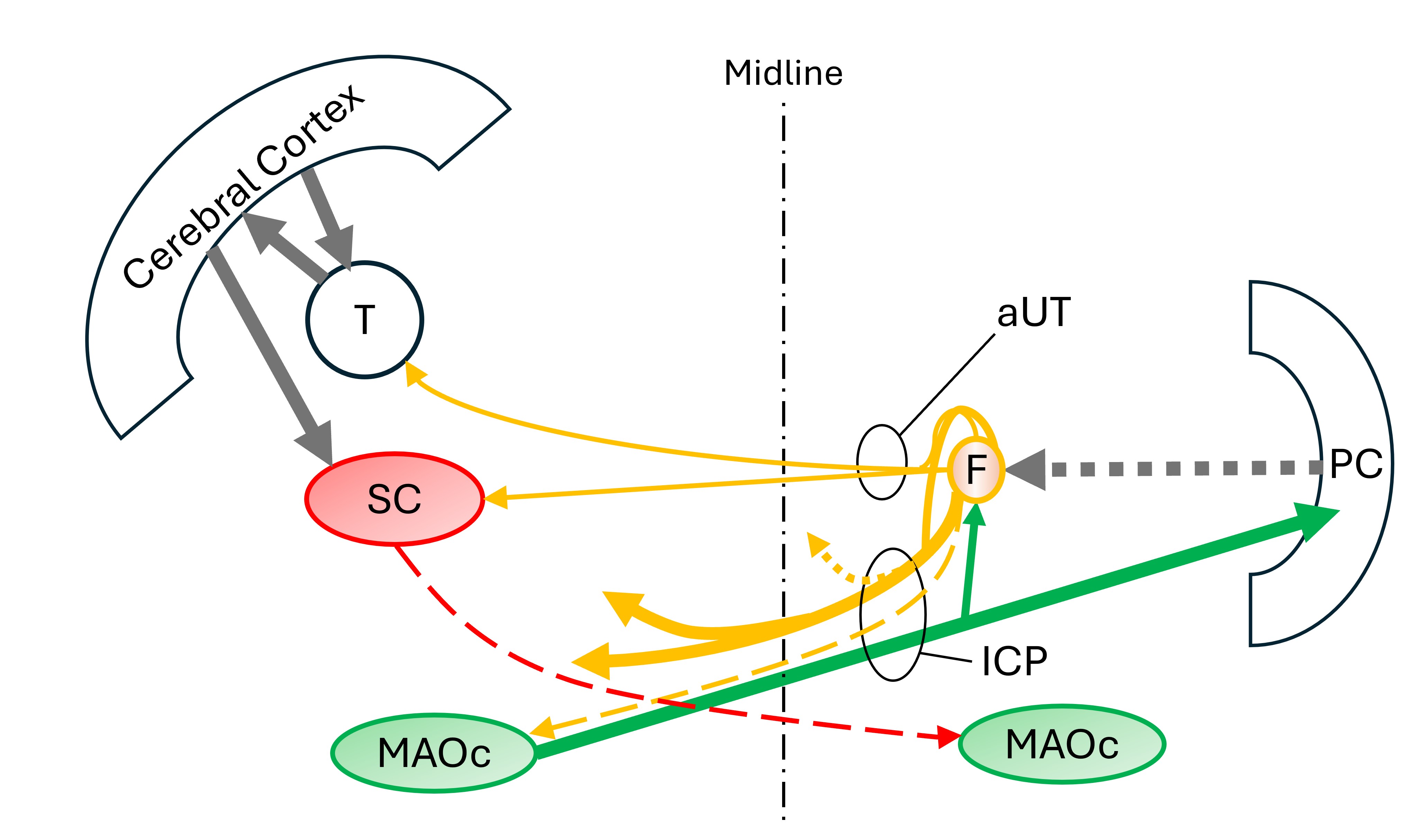

Supplement: Supplementary file 3 — (JPG 501 KB) [file 12311_2025_1903_MOESM3_ESM.jpg]
